# Impact of adhesive curing mode and dentin sealing on bond strength of CAD/CAM resin composite

**DOI:** 10.1038/s41598-025-31406-2

**Published:** 2025-12-19

**Authors:** Nermin Mohieldin Abdelmohssen, Mohamed Fouad Haridy, Sara Adel Botros

**Affiliations:** 1 Restorative Dentistry Department, Faculty of Dentistry, The British University in Egypt, Cairo, 11837 Egypt; 2 Conservative Dentistry Department, Faculty of Dentistry, Cairo University, Giza, 12613 Egypt

**Keywords:** CAD/CAM composite block, Universal adhesive, Curing mode, Dentin sealing, Microtensile bond strength, Adhesive dentistry, Biomedical materials, Nanoparticles

## Abstract

The aim of this study was to investigate influence of curing mode of universal adhesives and dentin sealing approach on microtensile bond strength (MTBS) of CAD/CAM composite to dentin. Occlusal surfaces of 36 human molars were ground to expose flat dentin and randomly assigned to 6 groups according to: (1) Adhesive curing mode [light-cured/LC (One coat 7 Universal, COLTENE); dual-cure/DC (One coat 7 Universal/Dual-cure Activator, COLTENE); and self-cure/SC (Palfique Universal Bond, Tokuyama); and (2) Dentin sealing approach (delayed sealing/DDS and immediate sealing/IDS). Following 1-week provisionalization, CAD/CAM composite blocks (Brilliant Crios, COLTENE), 4 mm^2^, were cemented over conditioned dentin surfaces. After 24 h, bonded assemblies were thermo-cycled for 5000 cycles. Specimens were tested for MTBS. Failure mode was analyzed under stereomicroscope. Data were statistically analyzed using ANOVA. DDS showed significantly higher MTBS values than IDS when using LC and DC adhesives. While, IDS produced significantly higher MTBS values than DDS when using SC adhesive. Predominant failure mode was adhesive in all groups, except for SC adhesive in IDS group, mixed failure was the predominant mode. Light- and dual-cured universal adhesives improved bond strength in delayed dentin sealing approach. Self-cure universal adhesive produced better bond strength when applied to immediately sealed dentin.

## Introduction

Continuous advancements in adhesive technology have significantly transformed the approach to restorative procedures throughout the last decades. Clinical efficacy of resin composite restorations predominantly depends on the adhesive system utilized to create a strong and durable bond with tooth structure^1^. Nevertheless, various issues may arise compromising the performance of adhesives. The infiltration of bacteria into the bond interface can lead to post-operative hypersensitivity, secondary caries, marginal discoloration, and a reduction in the longevity of the restoration^2^. Thus, composite restorations necessitate an optimal bond with dentin and enamel^3,4^.

Current adhesive technology aims to simplify the bonding procedures by reducing application steps, rendering clinical application shorter in time and less technique-sensitive. The so-called ‘universal’ or ‘multimode’ adhesives were introduced to serve this purpose^5,6^. These adhesives could be applied in different adhesive strategies as selective etching, etch-and-rinse and self-etch modes. They can also be used in bonding to ceramics, indirect resin composites and metal alloys^5,6^. However, studies have addressed chemical incompatibility issues between these adhesive systems and self- or dual-cure resin composites. When such combination is used, acidic monomers constituted in simplified adhesives react with tertiary amines of resin composites leading to unfavorable outcomes^7^.

In general, resin-based substances as adhesive systems attain their ultimate mechanical properties through a polymerization reaction. Appropriate polymerization of dental adhesives ensures better retention and marginal sealing and reduces nanoleakage and recurrent decay; thereby achieving sustained clinical effectiveness^8,9^. However, certain clinical scenarios impede access to light energy from light-curing units as when bonding indirect restorations. In such cases, light intensity reaching resin cement could be strongly attenuated, thereby negatively affecting its degree of conversion^10^. Consequently, dual-cure adhesive systems containing a ternary catalyst were introduced to the market to circumvent compatibility issues in case of light attenuation^11^. Recently, a novel self-curing system was launched to address the challenge of curing in the absence of light by substituting traditional benzoyl peroxide/tertiary amine system with a more stable catalytic one that endures strongly acidic conditions^12^.

Computer-assisted design and milling of prosthetics surpass traditional restorations in terms of precision, aesthetics, durability, and production efficiency^13^. CAD/CAM composite exhibits several key benefits over alternative CAD/CAM restorations: (1) It is less rigid than ceramics, minimizing clinical wear on opposing enamel. (2) It is easier to mill and repair. (3) It has lower brittleness, reducing likelihood of catastrophic failure and chipping during manufacturing. (4) It offers superior marginal integrity and compatibility with milling equipment^13^.

Immediate dentin sealing (IDS) denotes the application of a dentin bonding agent onto freshly prepared dentin for indirect restorations^14^. IDS offers numerous advantages, including protection of freshly cut dentin from contamination to enhance resin infiltration, minimizing post-operative hypersensitivity, reducing anesthesia requirements, and improving bond strength through hybrid layer maturation^14^.

Based on the aforementioned considerations, this study aimed to assess the effect of curing mode of different universal adhesive systems and dentin sealing approach on microtensile bond strength of a CAD/CAM composite block to dentin. The null hypothesis tested was that neither adhesive curing mode nor dentin sealing approach would influence microtensile bond strength of CAD/CAM composite block to dentin.

## Materials and methods

### Materials

All products’ description, composition, manufacturer and lot number are summarized in Table (1).

**Table 1:** Materials’ description, composition, manufacturer and lot number.

Product	Description	Composition	Manufacturer		Lot no.
One coat 7 Universal	Light-cure universal adhesive	10-MDP, Methacrylates, photoinitiator, HEMA, fillers, ethanol, water. (pH = 2.0–2.8)	Coltène/Whaledent AG, Altstätten, Switzerland	L27920	
One coat 7 Dual-cure Activator	Dual-cure activator	Ethanol, water, activator.	Coltène/Whaledent AG, Altstätten, Switzerland	L57700	
Palfique Universal Bond	Self-cure universal adhesive	**-Bond A**: Phosphoric acid monomer, MTU-6, HEMA, Bis-GMA, TEGDMA, Acetone. **-Bond B**: y-MPTES, borate, peroxide acetone, isopropyl alcohol, water. (pH = 2.2)	Tokuyama Dental, Tokyo, Japan	161E01	
Brilliant Crios	Reinforced composite CAD/CAM block	Cross-linked methacrylates (Bis-GMA, BIS-EMA, TEGDMA), 71 wt% barium glass, silica particle.	Coltène/Whaledent AG, Altstätten, Switzerland	K56598	
N-etch	Etching gel	37% phosphoric acid	Ivoclar Vivadent Schaan, Liechtenstein	Z03XMT	
Brilliant Flow	Nanohybrid flowable composite	-**Matrix**: Bis-GMA, TEGDMA, UDMA. -**Filler**: dental glass, amorphous silica.	Coltène/Whaledent AG, Altstätten, Switzerland	M06474	
Duo-Link Universal	Dual-cure self-etch resin cement	TEGDMA (5–20%), glass filler (50–80%), UDMA (5–15%), Bisphenol A diglycidyl methacrylate (5–30%)	BISCO, Schaumburg, IL, USA	2200007071	
Provitemp	Resin-based temporary cement	**Base and catalyst**: acrylate groups (70.87%), fluoride, chlorhexidine, potassium nitrate, dimethacrylates, silicon dioxide, catalysts.	ITENA, Villepinte, France	4236-10QTP	
Primma art	Self-curing composite	**-Base paste**: methacrylic monomers such as UDMA, TEGMA, co-initiators, silicon dioxide particles, inorganic pigments, barium aluminosilicate glass and stabilizers. -**Catalyst paste**: methacrylic monomers, dibenzoyl peroxide, aluminosilicate glass and stabilizers.	FGM, Joinville, SC, Brazil	100622	
Aquacare Al_2_O_3_ Powder	Abrasive powder	Aluminum oxide, 29 μm particle size.	Velopex international, Ospray Ltd, London, UK	220422	

## Methods

### Sample size calculation

Sample size calculation was conducted using G*Power 3.1.9.4 software based on a previous study by ***Sag et al. (2020)***^15^. Using an effect size of 0.78, 80% power and 5% significance level, 27 bonded assemblies were needed per each group. To compensate for pre-test failures, sample size was increased by 10% to 30 bonded assemblies retrieved from 6 molars for each group, hence, a total of 180 bonded assemblies from 36 molars.

### Study grouping

A total of 36 molars were randomly assigned to 6 groups (6 teeth per group) according to the study variables: (1) Adhesive curing mode [Light-cured universal adhesive (LS)- One Coat 7 Universal; Dual-cure universal adhesive (DC)- One Coat 7 Universal + Dual-cure Activator; and Self-cure universal adhesive (SC), Palfique Universal Bond]; and (2) Dentin sealing approach [delayed dentin sealing (DDS) or immediate dentin sealing (IDS)].

### Specimen preparation

A total of 36 freshly extracted sound human molars, free of cracks, caries and restorations were collected after obtaining ethical approval by the Research and Ethics Committee of the Faculty of Dentistry, The British University in Egypt, with approval no. 21–029. All methods were carried out in accordance with relevant guidelines and regulations as per the ethical board committee instructions. Informed consent was obtained from all subjects before teeth collection. Any residual soft and hard deposits were removed from roots using a scaler (Hu-Friedy Co., Chicago, IL, USA). Teeth were disinfected in 0.4% thymol for 24 h, and then stored in distilled water at room temperature. Each molar was mounted vertically in a PVC tube (1.5 cm diameter x 2 cm height) using self-cure acrylic resin (Acrostone, Cairo, Egypt) up to 2 mm below cemento-enamel margin. Occlusal enamel was horizontally sectioned to create flat mid-coronal dentin surfaces using microsaw mounted in a low-speed handpiece under copious water coolant. For a standardized dentin exposure, a predetermined mark on the external surface was made and verified through preoperative radiograph measurements.

Flat dentin surfaces were finished using extra-fine grit yellow-coded diamond bur (Mani, Tokyo, Japan) for 5 strokes in one direction to create a standardized smear layer. Each bur was discarded after 5 specimens. Specimens were ultrasonically cleaned using ultrasonic device (Vitasonic II, Vita Zahnfabrik H. Rauter GmbH & Co.KG, Bad Säckingen, Germany) in distilled water for 10 min. The following procedures were performed according to dentin sealing approach:

- **Immediate dentin sealing groups**: A light-cured universal adhesive (One Coat 7 Universal) applied to dentin surfaces, air-dried and light-cured for 20 s using LED light curing device (Elipar™ Deep Cure- L LED curing light, 3 M ESPE, Seefeld, Germany) with a light intensity > 1000 mW/cm². Dentin sealing was done by application of a 1 mm layer of nanohybrid flowable composite (Brilliant flow). Its thickness was verified by a graduated periodontal probe before light-curing. Initial curing for 10 s was performed, followed by final curing for 10 s through an oxygen barrier to prevent formation of oxygen inhibited layer. IDS layer was subsequently polished using rubber polishers (Diacomp Ultra, EVE Ernst Vetter GmbH, Keltern, Germany).
- **Delayed dentin sealing groups**: Dentin surfaces were left exposed without sealing.

All sealed and non-sealed dentin surfaces were then covered with temporary provisional restorations (Primmaart, FGM, Joinville, SC, Brazil) luted with a temporary cement (Provitemp, ITENA, Villepinte, France) in order to mimic the clinical scenario and stored in distilled water for one week in room temperature.

### Fabrication and surface treatment of CAD/CAM composite blocks

A total of 36 blocks were obtained from CAD/CAM reinforced composite blocks (Brilliant Crios). All blocks were cut using a low speed isomet saw under water coolant (Isomet 1000, Buehler Ltd., Lake Bluff, IL, USA) into 4 mm^2^ blocks. Before cementation, the cut surfaces were sandblasted with 29 μm with Al_2_O_3_ particles at 2 bar pressure at 10 mm distance for 20 s using Aquacare twin air abrasion unit (Velopex International, London, UK) according to manufacturer’s instructions^16^, followed by ultrasonic cleaning using the ultrasonic bath for 5 min in distilled water, and gentle air-drying.

### Cementation

After removal of provisional restorations, sealed dentin surfaces were sandblasted with 29 μm with Al_2_O_3_ particles at 2 bar pressure at 10 mm distance for 20 s using Aquacare twin. Then surfaces were etched for 30 s with 37% phosphoric acid etchant (N-etch, Ivoclar Vivadent, Opfikon, Switzerland) to eliminate any remnants of aluminum oxide particles embedded in the surface.

 The non-sealed dentin surfaces were ultrasonically cleaned using the ultrasonic device to remove any remnants of temporary cement. Dentin surfaces were then selectively etched for 5 Sect^17^. with 37% phosphoric acid etchant (N-etch, Ivoclar Vivadent, Opfikon, Switzerland) to eliminate any residual cement and condition the surface for adhesion before final cementation.

The different adhesive systems were applied as follows according to curing mode:

- **Group 1 (IDS) and 4 (DDS)**: a light-cured universal adhesive (One Coat 7 Universal) was applied to sealed and non-sealed dentin surfaces with the microbrush and agitated for 20 s, then gently air-thinned with oil-free compressed air for 10 s, and light-cured using LED light curing unit for 20 s.
- **Group 2 (IDS) and 5 (DDS)**: one drop of light-cured universal adhesive (One Coat 7 Universal) was mixed with one drop of dual-cure activator (One Coat 7 activator) in a dispensing well, applied to sealed and non-sealed dentin surfaces and agitated for 20 s, then gently air-thinned for 10 s and light-cured for 20 s.
- **Group 3 (IDS) and 6 (DDS)**: one drop from bottle A and B of self-cure universal adhesive (Palfique Universal Bond) was dispensed and mixed in a mixing well. The adhesive mix was applied to sealed and non-sealed dentin surfaces and agitated for 20 s, then gently air-thinned for 10 s and allowed to chemically cure following manufacturer’s recommendations.

For each group, a layer of the same respective adhesive was applied to the sandblasted surfaces of the composite blocks, air-thinned and either light-cured for 20 s or allowed to chemically cure^16^. A dual-cure resin cement (Duo-link universal, Bisco, Schaumburg, IL, USA) was dispensed directly onto the dentin surfaces through the provided automix tip and composite blocks were subsequently seated. A static load of 1 kg was applied over the blocks for 10 min using a specially-designed load device, in order to ensure a standardized and even pressure across all samples during the bonding procedure. This step helped achieve a uniform resin cement thickness, which is critical for minimizing variability among specimens; which thereby may potentially affect bond strength results.

Excess cement was removed immediately with a micro-brush and exposed margins were covered with glycerin gel after tac curing for 5 s from each side to ensure complete polymerization. Photo-polymerization was performed with the LED light curing unit for 20 s from occlusal, buccal and lingual directions. Finally, bonded specimens were stored for 24 h in 100% humidity at room temperature.

### Thermocycling

Specimens were subjected to thermocycling using a thermocycler (SD Mechatronik thermocycling, Julaba, Germany) for 5000 cycles (5–55 °C) for 15 s dwell time (simulating 6 months intraorally). Afterwards, all specimens were carefully inspected under a microscope to check for cracks or debonding.

### Micro-tensile bond strength testing (µ-TBS)

Specimens were sectioned along buccolingual and mesiodistal planes using a diamond disc (MTI Corporation, Richmond, CA, USA) in a low-speed micro-slicing machine (Isomet, Buehler Ltd., Lake Bluff, IL, USA) under water coolant, to produce beam-shaped specimens of approximately 1 mm^2^. Centralized 5 beams were retrieved from each specimen (*n* = 30 beams/group). Dimensions of the beams were verified with a digital caliper before testing. Beams were attached with cyanoacrylate glue to a gripping attachment mounted to a universal testing machine (INSTRON 2519 − 104, IL, USA). The beams were then pulled apart at a crosshead speed of 0.5/min until failure. Bond strength (MPa) was calculated by dividing failure load (N) by beam surface area (mm^2^).

### Failure analysis

After µ-TBS test, both dentin and composite sides of debonded beams were examined under stereomicroscope (Quattro ESEM, Thermo Fisher Scientific Ltd., CA, USA) at 50x magnification to determine failure modes. The failure modes were classified as follows:

#### Type 1

Cohesive failure in dentin.

#### Type 2

Adhesive failure at the resin cement-dentin interface.

#### Type 3

Mixed adhesive failure and cohesive failure in dentin.

#### Type 4

Cohesive failure in the resin cement.

#### Type 5

Mixed adhesive failure and cohesive failure in composite.

#### Type 6

Adhesive failure at the resin cement-composite interface.

### Statistical analysis

Statistical analysis was performed with R statistical analysis software version 4.3.1 for Windows (The R Project for Statistical Computing, New Zealand). The significance level was set at *p* < 0.05 within all tests. Numerical data were presented as mean and standard deviation (SD) values. Assumptions of normality were verified using Shapiro-Wilk’s. Effect of study variables on µ-TBS was analyzed using two-way ANOVA. Intergroup comparisons were conducted using one-way ANOVA followed by post-hoc Tukey’s test. P-values were adjusted for multiple comparisons using Bonferroni correction. Categorical data were presented as percentage values.

## Results

### I- Micro-tensile bond strength

#### Effect of study variables and their interaction

According to Two-Way ANOVA, the study variables (curing mode and dentin sealing approach) and their interaction significantly influenced micro-tensile bond strength (*p* < 0.001).

#### Effect of adhesive curing mode

Intergroup comparisons of micro-tensile bond strength mean values (MPa) within each dentin sealing approach using One-way ANOVA followed by post-hoc Tukey’s test are presented in Table (2). In IDS groups, there was a significant difference between curing modes (*p* < 0.001). SC group displayed the significantly highest µ-TBS value, followed by LC and DC groups; which were statistically similar. In DDS groups, there was a significant difference between curing modes (*p* < 0.001). DC group recorded the highest µ-TBS value, but did not significantly differ from that of LC group. While the significantly lowest µ-TBS was observed in SC group.

#### Effect of dentin sealing approach

Intergroup comparisons of micro-tensile bond strength mean values (MPa) within each curing mode using one-way ANOVA followed by post-hoc Tukey’s test are presented in Table (2). In LC and DC groups, DDS had significantly higher µ-TBS values than IDS (*p* < 0.001). While in SC group, DDS showed a significantly lower µ-TBS value than IDS (*p* < 0.001).

**Table 2:** Mean ± Standard Deviation (SD) of micro-tensile bond strength (MPa) in each group.

	Micro-tensile bond strength (MPa)			P-value
	LC (n=26/28)	DC (n=25/28)	SC (n=28/25)	
IDS	17.21±7.30^B^	13.74±6.49^B^	25.72±7.97^A^	<0.001*
DDS	35.90±6.75^A^	40.34±5.67^A^	14.12±8.44^B^	<0.001*
	<0.001*	<0.001*	<0.001*	

Values with different superscript letters within the same row are significantly different.

*: significant (*p* ≤ 0.05; (n = IDS/DDS): Total sample size after pre-test failures within each group.

### II- mode of failure

**Fig. 1:**
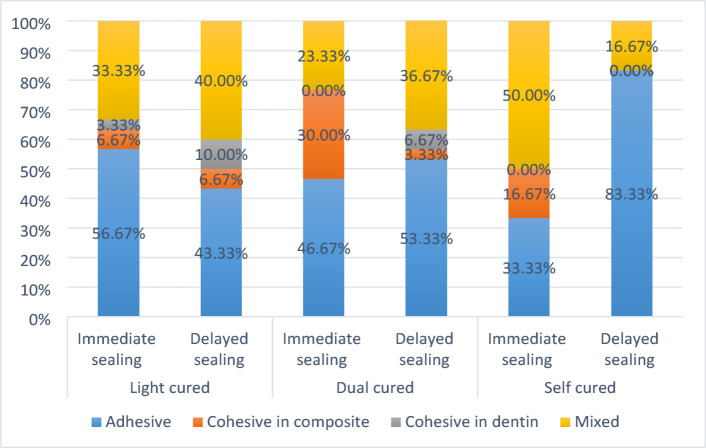
Stacked bar chart showing distribution of failure modes in each group.

Percentages of failure modes in each group are presented in Figure (1). In LC groups, the majority of samples of both dentin sealing approaches failed adhesively. Adhesive failures occurred in 56.67% of IDS group and 43.33% of DDS group. In DC groups, the overall failure mode analysis showed predominant adhesive failures; 46.67% in IDS and 53.33% in DDS groups. In SC groups, predominant failure of IDS samples was mixed type (50%); while the predominant failure in DDS samples was adhesive type (83.33%).

## Discussion

Indirect partial restorations have represented a more convenient option than direct composite restorations to restore extensive cavities, such as inlay and onlay restorations^18–20^. Today, recent advances in adhesives and resin cements have made indirect restorations become more widely used^18^. While optimal adhesion to enamel can be successfully achieved, bonding to dentin remains difficult due to its complex and heterogenous composition^1,4^. The treatment of hard dental tissues between preparation and final delivery plays a vital role in the success of indirect adhesive restorations^4^.

Adhesive systems acquire their final properties through a polymerization reaction, either light-curing, self-curing (chemically-activated reaction) or dual-curing where both curing mechanisms take place. The goal of this dual mechanism is to enhance polymerization reaction and increase degree of conversion, particularly in areas inaccessible to light source^21^. To circumvent compatibility issues with self- and dual-cure composites, dual-cure activators have been developed for use with simplified adhesives. These co-initiators are typically composed of sodium sulfinate salts which are mixed with the adhesive prior to application. These salts react with acidic monomers, to prevent inactivation of tertiary amines in resin composites^21^.

Milled composite restorations have several advantages over other machinable ceramic restorations because they exhibit less hardness and stiffness, so they are easy to mill and repair and causes less wear to opposing enamel. Their low brittleness results in less chipping and cracking during fabrication; hence higher marginal quality^13^.

Immediate dentin sealing has been recommended to be applied after tooth preparation to exposed dentin before taking an impression^14^. This protocol would ensure better infiltration to freshly cut, uncontaminated dentin and patent dentinal tubules. Additionally, this technique would prevent bacterial leakage and dentin hypersensitivity during the provisional stage^14,22^. Furthermore, by applying a flowable composite over a low film-thickness adhesive, it acts as a stress absorber and maintains the integrity and durability of the adhesive interface. On the other hand, delayed dentin sealing has drawbacks, as residual temporary cement might remain on the surface. Hence, final restorations are commonly bonded to contaminated dentin, which could hinder proper hybridization and reduce bond strength^23^.

In the current study, a standardized protocol was carefully followed for all specimens to minimize variability in the results. The depth of the exposed dentin, storage medium, smear layer creation, and testing parameters were kept consistent throughout the study.

The choice of storage medium is critical, as it should preserve dentin’s physical and mechanical properties without alterations, while also providing effective disinfection. In the present study, specimens were immersed in 0.1% thymol solution for 24 h to ensure microbial decontamination, followed by storage in distilled water for the remainder of the experimental period. It has been reported that disinfecting solutions may reduce dentin moisture and adversely affect the bond strength of resin composites. In contrast, distilled water, although lacking disinfecting properties, has been shown to maintain dentin moisture, microhardness, and bond strength stability^24^.

The microtensile bond strength (µTBS) test is considered one of the most reliable methods for evaluating the performance of contemporary adhesive systems, as it promotes a uniform stress distribution within the adhesive interface and minimizes stress-concentrating factors and voids. Previous studies have shown that varying the cross-head speed during µTBS testing did not influence bond strength values, likely due to the small specimen dimensions^25,26^.

Thermocycling was adopted in the study in order to mimic the thermal stress that restorative materials and teeth are subjected to due to consuming beverages and food and to speed up the simulated intraoral ageing process^27^.

Regarding dentin sealing protocol, our findings showed that delayed dentin sealing produced significantly higher bond strength values than immediate dentin sealing when using light- and dual-cure universal adhesives. Hence, the tested null hypothesis is rejected. These findings might be due to the presence of 10-MDP functional monomer component in One coat 7.0 universal adhesive which improved bond strength to exposed dentin surface of DDS groups through chemical bonding potential to hydroxyapatite, along with resin infiltration and resin tags formation. 10-MDP is one of the few monomers that has been shown to bond chemically to calcium found in hydroxyapatite (Ca_10_[PO4]_6_[OH]_2_) through ionic bonding^5^. The resulting MDP-calcium salts are hydrolytically stable and deposit in self-assembled nano-layers on dentin surface. Whereas in IDS groups, the adhesive was applied over the flowable composite sealing the dentin^3,5^. Phosphate groups of 10-MDP are able to chemically interact with hydroxyl groups contained in resin composite materials^3,8^. Since flowable composite used in this study for IDS does not constitute of zirconia fillers, the functional monomer has been of no benefit in such a case.

These conclusions are in accordance with the findings of a previous study (***Escobar et al.***,*** 2023)***^6^ reporting a relatively high dentin bond strength of universal adhesives with 10-MDP to direct composite regardless of the application mode, owing to its chemical bonding capacity to tooth structure, allowing for a stable and durable bond to dentin substrate. Additionally, adhesives containing 10-MDP monomer are less hydrophilic due to the longer molecular chain, resulting in stronger bonding to dentin. The interaction between 10-MDP and hydroxyapatite form nanolayers, rendering the adhesive more hydrophobic.

However, our results are not in line with those of a previous study ***(Sag et al.***,*** 2020)***^15^ which claimed that immediate dentin sealing significantly increased bond strength of CAD/CAM composite restoration to dentin. Noting that a 10-MDP-based universal adhesive was applied over IDS coating during cementation procedure of the indirect restoration in their study. ***Magne et al.***,*** (2007)***^22^ stated that IDS protocol improved MTBS to indirect restorations. The effective adhesion between immediate dentin sealing layer and fresh resin cement was justified by the presence of unreacted methacrylate groups and residual free radicals in the adhesive layer forming Van der Waals interactions (intermolecular forces); besides micromechanical retention resulting from air-borne particle abrasion. This disagreement with the above-mentioned studies might be due the differences in methodologies, such as types of adhesive system used for IDS and cementation, resin cement and indirect restoration, as well as absence of thermocycling. On the other side, ***Rozan et al. (2020)***^19^ reported that the application of a resin coating over dentin, whether a one-step self-etch adhesive or a combination of a two-step self-etch adhesive and a flowable composite, positively affected bond strength to CAD/CAM composite block. However, the effect of resin coating was relying on the type of resin cement used.

While for the self-cure universal adhesive, immediate dentin sealing displayed a significantly higher bond strength compared to delayed dentin sealing. Therefore, the null hypothesis investigated in this study is rejected. This outcome could be justified by the presence of a new silane coupling agent (γ-MPTES) in the formulation of Palfique universal bond, which has a methacryl group that co-polymerizes with monomers in uncured resin-based materials, and a hydrolyzable alkoxy groups which react with hydroxyl groups in the silica fillers of resin composite forming oxygen bridges. Bonding between the adhesive and the aged resin composite requires free radicals and unreacted methacrylate groups, which could be sparsely present in the aged flowable composite coat. For this reason, silane contained in the self-cure adhesive might have contributed to the higher bond strength results in IDS group. Silane has been mainly recommended to be used in case of repairing aged composite containing silica as a filler^28^. Hence, the methacrylate group of γ-MPTES contained in the adhesive formed covalent bonds with the resin cement from one side, and the alkoxy group reacted with the exposed amorphous silica fillers contained in the flowable composite coat from the other side. Silane could have also enhanced wettability of the adhesive over the irregularities created from sandblasting the flowable composite surface with aluminum oxide^29^, which improved micro-mechanical interlocking of composite-adhesive bonded interface^30^.

***Escobar et al. (2023)***^6^ reported lower bond strength results to dentin when using Palfique self-cure universal adhesive, which are in agreement with our findings concerning reduced bond strength values when delayed dentin sealing was adopted with the self-cure universal adhesive. They suggested the reason to be that isopropyl alcohol solvent contained in the adhesive increased water sorption and solubility compared to tested ethanol-based universal adhesive (One Coat 7 universal adhesive). Due to low vapor pressure of isopropyl alcohol, more time is needed for its evaporation compared to ethanol, which is present in the other adhesive (One Coat 7), delaying its polymerization. Also, the presence of HEMA, a hydrophilic monomer, in Palfique self-cure adhesive might have increased its water absorption during storage and thermocycling; and lowered the vapor pressure of water present as a co-solvent in the adhesive formulation as well, thereby hindering good solvent evaporation^31^. Residual water in adhesive and hybrid layers was claimed to be responsible for producing localized domains of incomplete monomer polymerization^32^. Thus, the extent of polymerization might vary along the hybrid layer, which could cause significant differences in the quality of hybrid layer at different regions. Moreover, inferior polymerization efficiency of HEMA itself has been found to compromise the mechanical properties of HEMA-containing adhesives, hence affecting bonding quality^32^.

As for the failure mode, the majority of the samples in light- and dual-cured universal adhesives showed predominant adhesive failure pattern in immediate dentin sealing groups. While the percentage of mixed failures increased in delayed dentin sealing groups. On the contrary, self-cure universal adhesive showed a predominantly adhesive failure in delayed dentin sealing group, and mostly mixed failures in immediate dentin sealing group. These observations largely reflect the bond strength results of this study. Adhesive failure between dentin-flowable composite interface was mostly observed in delayed dentin sealing group of the self-cure universal adhesive; while adhesive failure between flowable composite-resin cement interface were mostly observed in immediate dentin sealing group of light- and dual-cure universal adhesives in our study. In former studies, the weakest bond was at the resin coating-resin cement interface when immediate dentin sealing approach was adopted^33,34^. ***Rozan et al. (2020)***^19^ also reported that even if resin coating did not enhance bond strength, adhesive failure at dentin interface was unlikely found in groups with resin coating. This finding indicated that resin coating protected dentin surface when a CAD/CAM composite restoration was debonded or fractured.

To sum up, the chemical composition of adhesive systems, regardless of curing mode, can influence their bonding performance with different dentin sealing approaches. Clinicians should therefore consider the material formulation when selecting adhesives for immediate or delayed dentin sealing.

The main limitations of the present study may include the in-vitro nature of the study, which does not fully replicate the complex oral environment and its dynamic conditions such as temperature and humidity fluctuations and cyclic loading. Additionally, the location and orientation of dentinal tubules may still influence bonding performance despite the standardized preparation protocols. Further investigations should focus on finding the proper combination of an adhesive system and a low-viscosity hydrophobic resin that can be used to achieve an optimal protocol for immediate dentin sealing. Also, additional studies should be conducted to evaluate the effect of different adhesive systems with varying constituents, adhesive modes, resin cement and indirect restoration types, surface conditioning protocols, cavity configurations on bonding performance of indirect composite restorations to dentin.

## Conclusions

Under the conditions of the present study, the following conclusions could be derived:

1. Light- and dual-cured universal adhesives showed an improved bond strength with delayed dentin sealing protocol.
2. Self-cure universal adhesive enhanced bonding potential to immediately sealed dentin.
3. Light- and dual-cured universal adhesives showed predominant adhesive failure in both delayed and immediate dentin sealing.
4. Self-cure universal adhesive showed predominant adhesive failure pattern with delayed dentin sealing, while predominant mixed failure with immediate dentin sealing.
5. Chemical composition of adhesive systems, regardless of curing mode, demonstrated a potential effect on their performance with different dentin sealing approaches.

## Data Availability

All data analyzed in the current study are available in the article.
